# Combined use of Diane-35 and metformin improves the ovulation in the PCOS rat model possibly via regulating glycolysis pathway

**DOI:** 10.1186/s12958-020-00613-z

**Published:** 2020-06-03

**Authors:** Shun Zhang, Haoyan Tu, Jun Yao, Jianghua Le, Zhengxu Jiang, Qianqian Tang, Rongrong Zhang, Peng Huo, Xiaocan Lei

**Affiliations:** 1 Department of Reproductive Medical Center, The Affiliated Hospital of Guilin Medical University, Guilin, 541001 China; 2 School of Basic Medical Sciences, Zunyi Medical University, Zunyi, 563000 China; 3 School of Public Health, Guilin Medical University, Guilin, 541004 China; 4 Clinical Anatomy & Reproductive Medicine Application Institute, Department of Histology and Embryology, University of South China, Hengyang, 421001 China

**Keywords:** PCOS, Diane-35, Metformin, Apoptosis, Glycolysis, SIRT1

## Abstract

**Background:**

Polycystic ovary syndrome (PCOS) is a complex endocrine and metabolic disease with unknown pathogenesis. However, the treatment of Diane-35 combined with metformin can improve the endocrine and ovulation of PCOS. In this study, we investigated the effects of Diane-35 combined with metformin (DM) treatment on ovulation and glucose metabolism in a PCOS rat model.

**Methods:**

Sprague Dawley rats were divided into 3 groups, control group, model group (PCOS group) and Diane-35 combined with metformin (PCOS + DM group). The mRNA expression levels were determined by qRT-PCR. The hormone levels were determined by enzyme-linked immunosorbent assay. Immunostaining detected the protein levels of lactate dehydrogenase A (LDH-A), pyruvate kinase isozyme M2 (PKM2) and sirtuin 1 (SIRT1) in the ovarian tissues. TNUEL assay was performed to determine cell apoptosis in the PCOS rats. The metabolites in the ovarian tissues were analyzed by liquid chromatography with tandem mass spectrometry.

**Results:**

PCOS rats showed an increased in body weight, levels of luteinizing hormone and testosterone and insulin resistance, which was significantly attenuated by the DM treatment. The DM treatment improved disrupted estrous cycle and increased the granulosa cells of the ovary in the PCOS rats. The decreased proliferation and increased cell apoptosis of granulosa cells in the ovarian tissues of PCOS rats were significantly reversed by the DM treatment. The analysis of metabolics revealed that ATP and lactate levels were significantly decreased in PCOS rats, which was recovered by the DM treatment. Furthermore, the expression of LDH-A, PKM2 and SIRT1 was significantly down-regulated in ovarian tissues of the PCOS rats; while the DM treatment significantly increased the expression of LDH-A, PKM2 and SIRT1 in the ovarian tissues of the PCOS rats.

**Conclusion:**

In conclusion, our study demonstrated that Diane-35 plus metformin treatment improved the pathological changes in the PCOS rats. Further studies suggest that Diane-35 plus metformin can improve the energy metabolism of the ovary via regulating the glycolysis pathway. The mechanistic studies indicated that the therapeutic effects of Diane-35 plus metformin treatment in the PCOS rats may be associated with the regulation of glycolysis-related mediators including PKM2, LDH-A and SIRT1.

## Introduction

Polycystic ovary syndrome (PCOS) is a complex, common endocrine condition and metabolic disease affecting reproductive aged women with a reported prevalence of 8–13% in the world, and PCOS is the main cause of primary infertility [1]. The clinical manifestations of PCOS include hyperandrogenism (hirsutism, acne), irregular menstruation, ovulatory dysfunction, reproductive endocrine hormone disorder, insulin resistance and dyslipidemia. Dysfunction of the hypothalamic–pituitary–ovarian axis has been regarded as the main cause of PCOS [2]. In PCOS women, the gonadotropin-releasing hormone pulse frequency is increased, which favors increased luteinizing hormone (LH) secretion over that of follicle-stimulating hormone (FSH). However, there is relative deficit in FSH secretion, which often results in impaired and arrested follicular development and reduced aromatase activity, thereby resulting in ovarian follicular atresia and hyperandrogenemia in PCOS women [3]. In addition, high insulin levels in PCOS patients could increase the expression of LH, which could promote the secretion of androgen from the ovary and adrenal glands. The interaction between increased levels of insulin and LH can cause an increase in atresia follicles in the ovary, which may lead to development of PCOS [4]. Diane-35 (composed of 2 mg cyproterone acetate and 35 μg ethinyl estradiol) plus metformin are the two commonly used drugs in clinical to treat PCOS, which have the advantages of improving glucose metabolism, modifying the insulin resistance state, reducing testosterone levels in serum and restoring ovulation of the ovary in patients with PCOS [5]. However, the current research on the treatment of PCOS patients is at its preliminary stage; while the mechanism of how to improve folliculogenesis is still unclear.

There is growing evidence showing that granulosa cell energy metabolism disorder is an important cause of abnormal follicles and fertility decline in females. The main energy metabolism of normal folliculogenesis is that granulosa cells take up glucose from surrounding tissues via the glucose transporter (GLUT) on their cell membranes, then glucose producing pyruvic acid and lactic acid through the glycolysis pathway [6, 7]. A large number of studies has shown that pyruvic acid and lactic acid are important factors in maintaining follicular growth and development [8]. Harris et al. found that follicles in patients with PCOS require more pyruvate to maintain follicle growth than normal female [9]. In vitro results show that testosterone and insulin stimulation can reduce lactic acid content in granulosa cells [10]. The above studies indicate that the follicles of patients with PCOS require more pyruvate and lactic acid to stimulate follicle to develop normally on the one hand, and on the other hand, high androgen and insulin reduce the content of lactic acid. However, whether Diane-35 plus metformin treatment could restore the dysfunction of granulosa cell energy metabolism in PCOS development remain elusive.

In this study, we investigated the effects of Diane-35 combined with metformin treatment on ovulation in a PCOS rat model. Further studies were performed to determine the effects of the combined treatments on energy metabolism in the ovary. In addition, the ovarian glycolysis-related rate-limiting enzymes and sirtuin 1 (SIRT1) expression was determined to elucidate the mechanistic actions of the combined treatment in restoring the dysfunction of energy metabolism in the PCOS ovary. The present study could provide a new insight into understanding the mechanistic actions underlying therapeutic effects of Diane-35 combined with metformin treatment on PCOS.

## Materials and methods

### Animals and treatments

A total of 40 female Sprague Dawley (SD) rats (5 weeks old and 130–140 g) were purchased from the Laboratory Animal Center of the Third Military Medical University (Chongqing, China). The animals were randomly divided into a control group (10 animals) and a PCOS group (30 animals). The PCOS group was intragastric administration with letrozole [11] (Heng Rui Pharmaceutical Factory, Lianyungang, Jiangsu, China) dissolved in 1% (w/w) CMC and fed with high fat diet. Vaginal smear of SD rats was monitored after 1 week of modeling, and the establishment of the model was preliminarily judged to be successful or not according to the estrous cycle. Two rats with successful initial model were randomly selected for ovarian morphology and serum reproductive hormone analysis for verification. We divided SD rats successfully modeled into PCOS group (10 animals) and PCOS + Diane-35 plus metformin (DM) group (10 animals). The PCOS + DM group was treated as follows: Diane-35 and metformin were intragastric administration to SD rats up to 21 days. The PCOS group was drenched with 1% CMC up to 21 days. All the animal experiments were approved by the Animal Ethics Committee of First Affiliated Hospital of Guilin Medical University (No.GLMC202003153).

### Vaginal cytology

Vaginal smears were performed respectively for 14 d before the end of modeling and treatment. Briefly, the vagina was flushed with 35 μL normal saline (0.9% NaCl) for 2–3 times. The vaginal fluid (10–20 μL) was collected onto the glass slide. After air dry at room temperature, the slides were fixed with methanol followed by staining with the Wright’s–Gimsa (Solarbio, Beijing, China) according to the manufacturer’s protocol. The changes of vaginal epithelial cells were evaluated under a light microscope to determine the estrous cycle.

### Serum collection and measurement of hormone levels

At the end of the experiment, the rats were anesthetized by intraperitoneal injection of 10% chloral hydrate (0.2 ml/100 g). Blood was collected by cardiac puncture, and the serum was isolated by centrifuging at 2500 rpm for 20 min. The serum levels of testosterone and LH were determined by corresponding commercial enzyme-linked immunosorbent assay kits (Abcam, Cambridge, USA).

### Morphological analysis of ovary

The ovarian tissues were dissected and fixed in 4% paraformaldehyde overnight at 4 °C, embedded in paraffin, sectioned into 5-μm thick slices, deparaffinized and stained with hematoxylin and eosin. The morphology of the ovarian tissues was evaluated under a light microscope.

### Immunohistochemistry analysis of proliferating cell nuclear antigen (PCNA), lactate dehydrogenase a (LDH-A), pyruvate kinase isozyme M2 (PKM2) and SIRT1

Ovaries were fixed in 4% formaldehyde and embedded in paraffin; 5-μm thick slices were sectioned. The sections were permeabilized with 1% Triton X-100 in phosphate buffered saline (PBS) for 30 min at room temperature, boiled in 100 mM sodium citrate (pH 6.0) three times for 6 min each at 5-min intervals for antigen retrieval, and then incubated with 3% hydrogen peroxide for 30 min to remove endogenous peroxidase followed by blocking in 5% bovine serum albumin at room temperature for 1 h. The sections were then incubated overnight at 4 °C with primary goat polyclonal PCNA, LDH-A, PKM2 and SIRT1 antibodies in the blocking solution. Following three washes with 0.1% Tween-20 in PBS, the samples were incubated with rabbit anti-goat biotin-SP-conjugated antibody (1:100; SA00004–4, Protein Tech Group Inc.) in the blocking solution at room temperature for 45 min. The stained PCNA, LDH-A, PKM2 and SIRT1 proteins were visualized using the 3, 3-diaminobenzidine chromogen. The primary antibody replaced with normal goat IgG was served as a negative control. The stained sections were evaluated under a light microscope.

### Terminal Deoxynucleotidyl Transferase-mediated dUTP Nick end-labeling (TUNEL) analysis

Detection of the apoptotic cells in the ovarian granular cells was determined by TUNEL assay (Roche Diagnostic Systems, Branchburg, USA) by following the manufacturer’s instructions with modifications [12]. In brief, sections were dewaxed and rehydrated in water, and then treated with proteinase K and 3% hydrogen peroxide followed by incubating with the TUNEL reaction mixture in a humidified chamber at 37 °C. After that, the sections were incubated with peroxidase-conjugated anti-biotin antibody. The apoptotic cells were visualized using the 3, 3-diaminobenzidine chromogen. Haematoxylin was used as counterstaining.

### Quantitative real-time PCR (qPCR)

RNA extraction from the tissues was performed using TRIzol reagent (Takara, Dalian, China). Synthesis of cDNA was performed using the TransScript II One-Step gDNA Removal and cDNA Synthesis SuperMix kit (Transgen Biotech, Beijing, China) according to manufacturer’s protocol. Real-time PCR analyses for the gene expression level were performing on the Applied Biosystems 7500 Real-time PCR System (Applied Biosystems, Foster City, CA, USA). GAPDH was used as the reference control, and gene expression levels were calculated using comparative Ct method. The primer sequences were shown in Table 1.

**Table 1:** Primer sequences of the qRT-PCR analysis

Gene	Sequence (5′-3′)	Annealing Temperature	Product (bp)	GeneBank No
PCNA	F: GCTCCATCCTGAAGAAGGT	55 °C	121	NM-022381.3
	R: TGCACTAAGGAGACGTGAGA			
Bax	F: GAGACACCTGAGCTGACCTT	55 °C	104	NM-017059.2
	R: TCCATGTTGTTGTCCAGTTC			
Bcl-2	F: AGTACCTGAACCGGCATCT	55 °C	120	NM-016993.1
	R: CCGGTTACTATTCCTGGAGA			
Caspase-3	F: CCGGTTACTATTCCTGGAGA	55 °C	117	NM-017008.4
	R: TAACACGAGTGAGGATGTGC			
MCT4	F: CCTTCCTTCTCACCATCCT	55 °C	136	BC168146.1
	R: TCAGTGAAGCCATTGAAGAA			
MCT2	F: GGATTGGGATTTGGAAGTAT	55 °C	119	NM-003676.8
	R: AGAACTGGACAACACTCCAC			
GLUT1	F: TGGCTCCTCATGTCAGAGA	55 °C	125	AJ245935.1
	R: AGGATCTCCATGATGCTGTT			
HK	F: AGAGGCTACGGACAGAGATG	55 °C	121	NM-012734.1
	R: AGGAAGTCACCGTGTTCAGT			
PFK	F: GGCGTGTGTTCATTGTAGAG	55 °C	125	NM-017008.4
	R: CTTCAAGTCGTGGATGTTGA			
PKM	F: ACATCCTGTGGCTGGACTAT	55 °C	130	NM-053297.2
	R: TCCACTTCTGTCACCAGGTA			
LDH	F: GGTTGACAGTGCATACGAAT	55 °C	106	NM-017025.1
	R: CCGCCTAAGGTTCTTCATTA			
GAPDH	F: CCTCAAGATTGTCAGCAATG	55 °C	134	NM-017008.4
	R: CAGTCTTCTGAGTGGCAGTG			

### Liquid chromatography with tandem mass spectrometry (LC-MS/MS) analysis of ovarian metabolites

A total of 40 mg rat ovarian tissue for each sample was homogenized in 200 μL pre-cooled ultrapure water. The homogenized samples were incubated with 800 μL pre-cooled methanol/acetonitrile (1:1, v/v) by vortex mixing with ultrasound in ice bath for 20 min, followed by incubation for 1 h at − 20 °C and centrifuge at 14000 r/min for 4 min at 4 °C. The supernatant was collected and dried in the vacuo. For mass spectrometry, 100 μL of acetonitrile-water solution (1:1, v/v) was reconstituted, centrifuging at 14000 r/min for 4 min at 4 °C, and the supernatant was taken for analysis. Samples were separated by using an Agilent 1290 Infinity LC Ultra Performance LC System. Mobile phase: liquid A was 10 mM aqueous ammonium acetate solution, and liquid B was acetonitrile. The sample was placed in a 4 °C autosampler at a column temperature of 45 °C with a flow rate of 300 μL/min and an injection volume of 2 μL. The relevant liquid phase gradient was as follows: 0–18 min, B liquid linearly changes from 90 to 40%; 18–18.1 min, B liquid linearly changes from 40 to 90%; 18.1–23 min, B liquid is maintained at 90%. A quality control sample is set up for each experimental sample in the sample queue for the detection and evaluation of the stability and repeatability of the system; a standard mixture of energy metabolites is set in the sample queue for the correction of chromatographic retention time. Mass spectrometry was performed in negative ion mode by using a 5500 QTRAP mass spectrometer (AB SCIEX). The 5500 QTRAP ESI source conditions were as follows: source temperature 450 °C, ion Source Gas 1: 45, Ion Source Gas 2: 45, Curtain gas: 30, ion Spray Voltage Floating- 4500 V; multi-response monitoring mode detects the pair of ions to be tested. The peak area and retention time were extracted by using Multiquant software. Standardization of energy metabolites was used to correct retention time for metabolite identification.

### Statistical analysis

Data were analysed using SPSS 18.0 software (SPSS, Inc., Chicago, IL, USA). The experimental data are presented as the mean ± standard deviation. The unpaired Student’s t-test was used to analyze the comparison between the two groups. One-way ANOVA followed by Bonferroni’s multiple comparison tests was used for comparison among multiple groups. A *P* value less than 0.05 was considered to indicate a statistically significant result.

## Results

### Effect of DM treatment on body weight, hormone level, estrous cycle and ovary morphology in PCOS rats

**Fig. 1:**
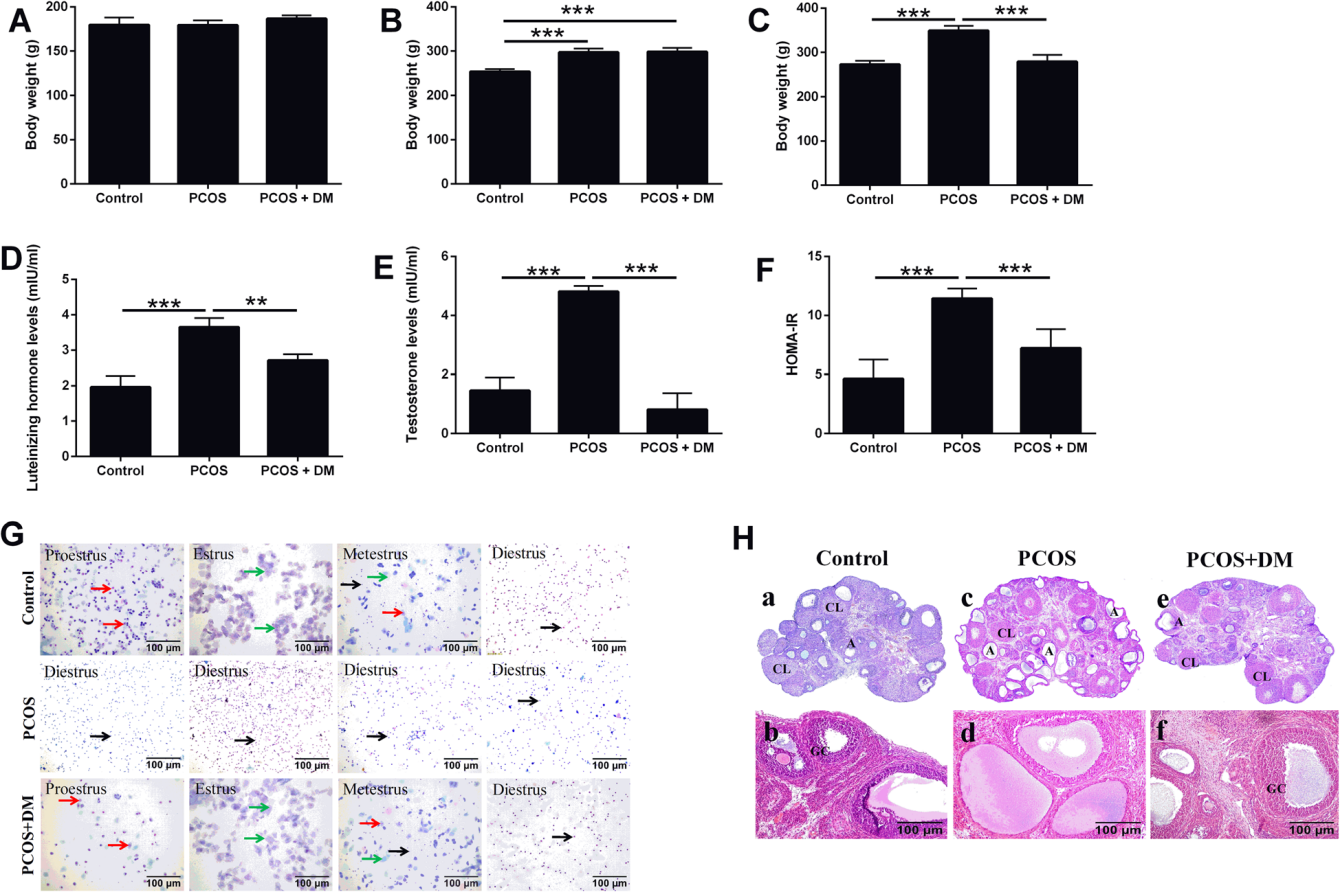
Effects of DM treatment on body weight, hormone levels, estrous cycle and ovary morphology in PCOS rats. **a** Body weight of the rats before the induction. **b** Body weight of the rats after PCOS modeling. **c** Effects of DM treatment on the body weight of the DM-treated rats. **d-f** Effects of DM treatment on the luteinizing hormone levels, testosterone levels and HOMA-IR in the PCOS rats. **g** Effects of DM treatment on the estrous cycle of the PCOS rats. **h** Effects of DM treatment on the ovary morphology in the PCOS rats. *N* = 6. Significant differences between groups were indicated as **P < 0.01 and ***P < 0.001

There was no significant difference in body weight before induction of PCOS (Fig. 1a). After the induction of PCOS in the rats, body weight in the PCOS group increased remarkably (Fig. 1b). DM treatment significantly reduced weight gain induced by PCOS (Fig. 1c). In addition, the levels of luteinizing hormone and testosterone, and HOMA-IR were significantly increased in the PCOS rats, which was markedly attenuated by the treatment with DM (Fig. 1d-f).

SD rats have an estrous cycle about 4–5 days. The vaginal smear results of the rats were shown in Fig. 1g. The vaginal smear in the early stage of estrus is mainly composed of nuclear epithelial cells with nuclei grayish red and cytoplasm grayish (the red arrows). Estrous phase is mainly composed of keratinocytes with flat shape in blue (the green arrows). White blood cells and nuclear epithelial cells are the main components at metestrus with keratinocytes occasionally. The diestrus phase is almost entirely white blood cells (the black arrows). In our study, we found that the control group had a complete estrous cycle. In the PCOS group, the estrous cycle disorder appeared successively on the 10th day after the high-fat diet and letrozole intragastric administration, almost at metestrus. In the PCOS+DM group, the estrous period gradually appeared at 14 days after treatment, and the complete estrous period appeared at 21 days (Fig. 1g). The morphology of ovary was observed by using hematoxylin-eosin staining. In the PCOS group, multiple follicles with cystic expansion appeared, which were vacuolated and disorganized in structure with corpus luteum occasionally seen and atresia increased. The number of granulosa cells decreased and the granulosa cells even disappeared. Some oocytes and radiating crowns were lost. In the PCOS+DM group, follicles of different stages and multiple luteal bodies were observed (Fig. 1h). The number of granular cell layers increased after DM treatment (Fig. 1h).

### Effects of DM treatment on the PCNA expression and apoptosis-related mediators expression in the ovary of PCOS rats

**Fig. 2:**
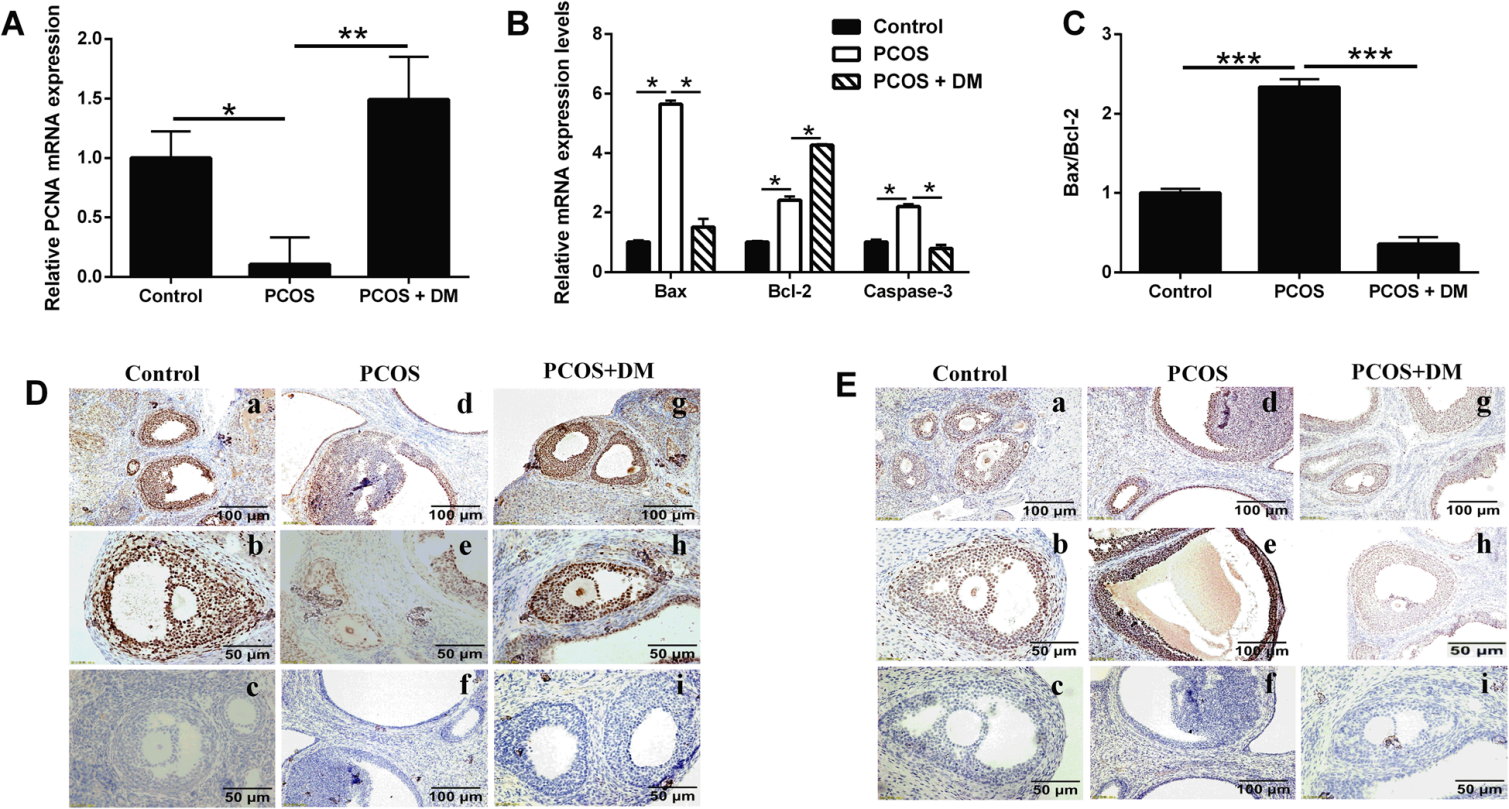
Effects of DM treatment on the PCNA expression and apoptosis-related mediators expression in the ovary of PCOS rats. **a** Effects of DM treatment on the PCNA mRNA expression levels in the ovarian tissues from PCOS rats. **b-c** Effects of DM treatment on the mRNA expression levels of Bax, Bcl-2 and caspase-3 mRNA expression levels in the ovarian tissues from PCOS rats. **d** IHC analysis of PCNA protein expression in the ovarian tissues of the PCOS rats after different treatments. **e** TUNEL analysis of apoptotic cells in the ovarian tissues of the PCOS rats after different treatments. N = 6. Significant differences between treatment groups were indicated as *P < 0.05, **P < 0.01 and ***P < 0.001

The mRNA expression of PCNA in the ovarian of the rats was analyzed by qRT-PCR. We found that PCNA expression in PCOS group was significantly lower than that in control group (Fig. 2a). The mRNA expression of PCNA in ovary increased significantly after the DM treatment (Fig. 2b). Furthermore, qRT-PCR was used to analyze the expression levels of apoptosis-related genes including Bax, Bcl-2 and caspase-3 in follicular granulosa cells. The results showed that the expression of Bax, Bcl-2 and caspase-3 in the ovary of PCOS group was significantly increased compared with the control group; while the increase in these apoptosis-related genes in the PCOS group was significantly attenuated by the DM treatment (Fig. 2b). The ratio of Bax/Bcl-2 in the PCOS group was significantly higher than that in the control group, which was significantly decreased after the DM treatment (Fig. 2c). The proliferation of ovarian granulosa cells was detected by immunohistochemistry. PCNA protein was expressed in follicular granular cells including granular cells from follicle walls and ovarian cumulus under the microscope in control group (Fig. 2d). In comparison with the control group, the number of PCNA-positive cells in PCOS group was significantly decreased (Fig. 2d). After the DM treatment, the protein expression of PCNA was enhanced significantly with a strong expression in granular cells from follicle walls and ovarian cumulus, which was similar to the control group (Fig. 2d). TUNEL assay was conducted to determine the apoptosis of granular cells in the ovary. Granulosa cells are stained in brown as apoptotic cells under the microscope. The color of nucleus of the negative control group was blue. The results showed that many brown particles were observed in the ovarian granular cells in the PCOS group and stained as TUNEL positive (Fig. 2e) but rare in the control group (Fig. 2e). After the DM treatment, the number of apoptosis of granular cells was decreased (Fig. 2e).

### Effects of DM on ovarian production of energy metabolism

**Fig. 3:**
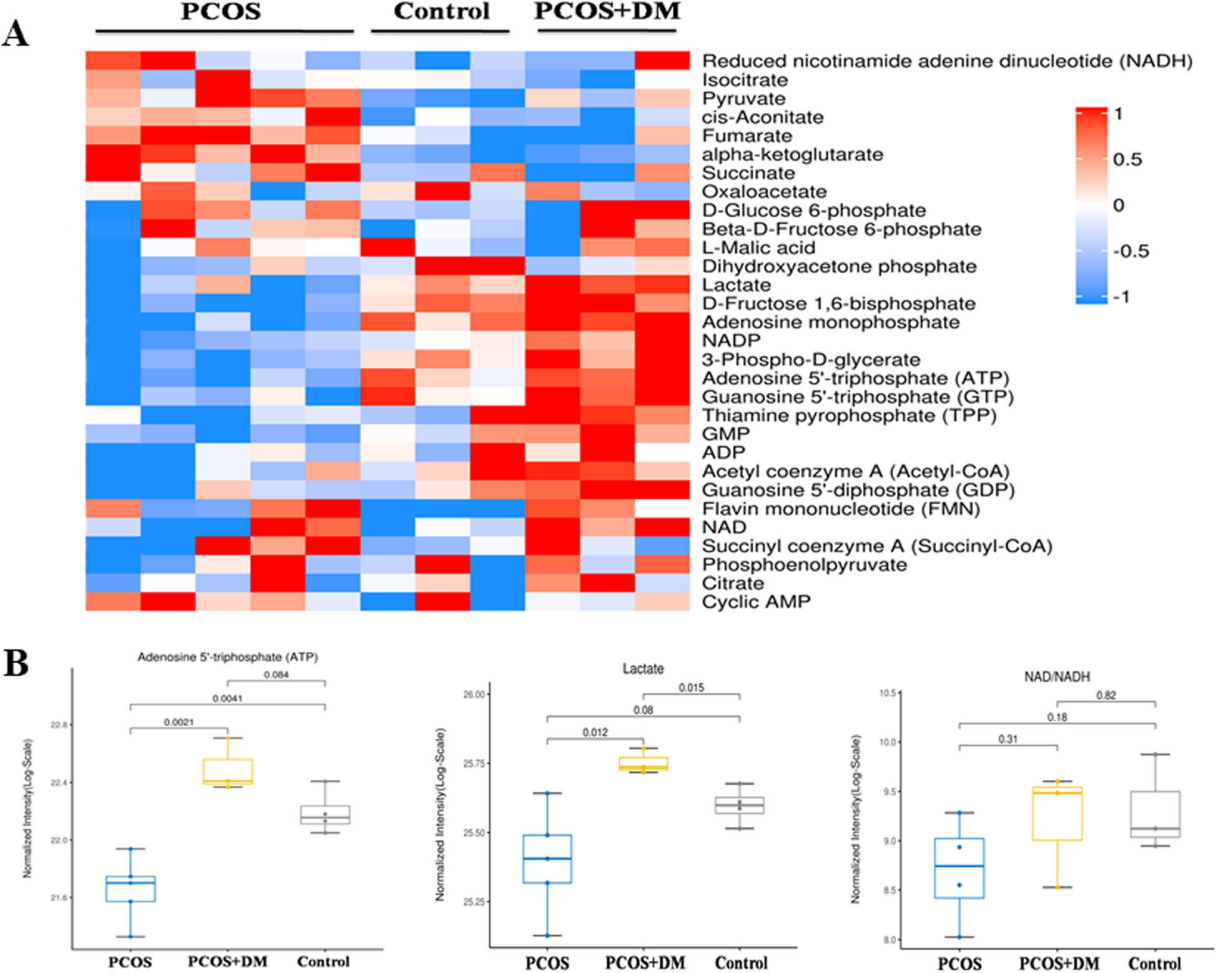
Effects of DM treatment on the metabolic mediators in ovarian tissues of PCOS rats. **a** Effects of DM treatment on the metabolic mediators in the ovarian tissues of PCOS rats by using the targeted metabolomics analysis. **b** Effects of DM treatment on the levels of ATP, lactate and NAD+/NADH levels in the ovarian tissues of PCOS rats after different treatments. N = 6

Ovarian energy metabolites were measured by multi-response monitoring. The levels of lactate, production of purine metabolism and production of oxidative phosphorylation in the ovary of PCOS+DM group were significantly increased compared with the PCOS and control group as illustrated in the heatmap (Fig. 3a), which suggested that combination therapy could improve follicular energy supply in PCOS rats. The ATP level in PCOS group was significantly lower than that in control and PCOS + DM group (Fig. 3b). In addition, DM treatment could significantly increase the content of ATP in ovaries of PCOS rats (Fig. 3b). The lactate in PCOS group was higher than that in control group, and the DM treatment significantly reduced the lactate levels in the PCOS rats (Fig. 3b). As for the changes of NAD^+^/NADH, there was no significant difference among three groups (Fig. 3b).

### Effects of DM treatment on ovarian glycolysis-related rate-limiting enzymes and SIRT1 expression

**Fig. 4:**
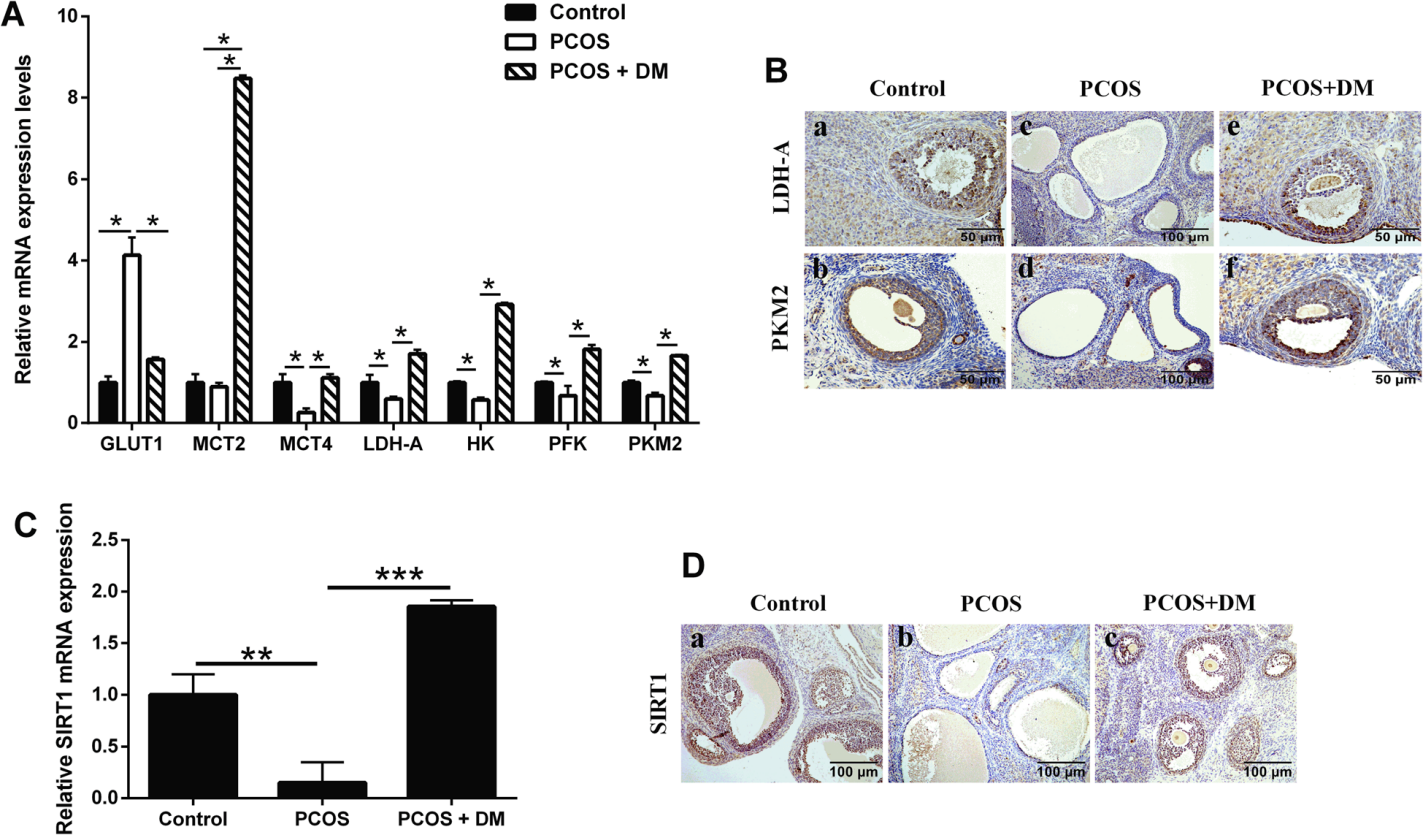
Effects of DM treatment on the PKM2, LDH-A and SRIT1 expression levels in the ovarian tissues from PCOS rats. **a** qRT-PCR analysis of GLUT1, MCT2, MCT4, LDH-A, HK, PFK and PKM2 in the ovarian tissues of PCOS rats after different treatments. **b** IHC analysis of LDH-A and PKM2 protein expression in the ovarian tissues of PCOS rats after different treatments. **c** qRT-PCR analysis of SIRT1 mRNA expression levels in the ovarian tissues of PCOS rats after different treatments. **d** IHC analysis of SIRT1 protein expression in the ovarian tissues of PCOS rats after different treatments. N = 6. *P < 0.05, **P < 0.01 and ***P < 0.001

The expression changes of glycolysis-related rate-limiting enzymes in ovary were analyzed by qRT-PCR. The expression of GLUT1 was significantly increased and hexokinase (HK) was significantly decreased in the PCOS group when compared to the control group. In addition, the expression levels of monocarboxylate transporter 4 (MCT4), LDH-A, phosphofructokinase (PFK) and PKM2 were all decreased in the PCOS group when compared to the control group (Fig. 4a). After the DM treatment, glycolysis-related rate-limiting enzymes were up-regulated significantly (Fig. 4a). Furthermore, the expression of LDH-A and PKM2 were detected by immunohistochemistry. LDH-A and PKM2 proteins were expressed in follicular granular cells in control group (Fig. 4b). In comparison with the control group, the number of LDH-A- and PKM2-positvie cells in PCOS group was significantly decreased (Fig. 4b). After DM treatment, the expression of LDH-A and PKM2 was significantly enhanced in the PCOS rats (Fig. 4b). The expression of SIRT1 was detected by qRT-PCR and immunohistochemistry. The mRNA expression of SIRT1 in ovary from PCOS group was lower than that in control group, while the mRNA expression of SIRT1 was significantly increased after combination therapy compared to the PCOS group (Fig. 4c). SIRT1 protein was expressed in follicular granular cells including ovarian cumulus under the microscope in control group (Fig. 4c). In comparison with the control group, the number of positive cells of SIRT1-positive cells in PCOS group was significantly decreased (Fig. 4d). After DM treatment, the protein expression of SIRT1 was significantly enhanced (Fig. 4d).

## Discussion

Currently, high insulin resistance in PCOS patients is a hot topic in the field of reproductive endocrinology and insulin resistance appears to be the fundamental key factor within the pathophysiology of PCOS [13]. In order to fully understand the pathogenesis of PCOS in vivo studies, it is important to establish an animal model that is close to human polycystic ovarian disease changes. Although letrozole treated SD rats exhibited many metabolic features of human PCOS and have been commonly used as an animal model for PCOS, there was no change in insulin sensitivity or lipid metabolism [11]. Thus, we established the PCOS-IR rat model by treating the animals with letrozole and high-fat diets. The PCOS-IR rats showed increased body weight, elevated levels of luteinizing hormone and testosterone, and increased HOMA-IR; the The ovaries of PCOS-IR rats demonstrated a PCOS-like appearance, which contained large follicular cysts with poorly developed granulosa cells and many atretic follicles, which were similar to the pathological changes of human PCOS-IR [14]. Therefore, the rat model of letrozole for 30 days with high-fat diet can not only study polycystic changes of PCOS ovary, but also study metabolic diseases such as insulin resistance, which is an ideal model for studying the pathogenesis of PCOS-IR.

Diane-35 is composed of 2-mg cyproterone acetate and 35-μg ethinyl estradiol, which is widely used for the management of PCOS patients with hyperandrogenism [15]. It works by blocking the effects of androgens such as testosterone and by activating the progesterone receptor. The function of metformin is commonly described for the modulation of insulin sensitivity and glucose metabolism in PCOS women [16]. Combination of Diane-35 and metformin treatment could decrease the BMI and LU levels, improve the condition of hyperandrogenemia and insulin resistance in PCOS patients [5]. In the dihydrotestosterone-induced PCOS rats, Diane-35 was found to be effective to restore the reproductive functions [17]. Consistently, in the present study, we found that combination of Diane-35 and metformin treatment reduced body weight, decreased the levels of testosterone and luteinizing hormone and insulin resistance, and also restored the estrous cycle and ovulation in the PCOS rats, indicating that the combined treatment improved the PCOS complications.

In a further study, we found that there is a decrease in the granulosa cell proliferation (as determined by the measurement of PCNA expression) and an increase in the granulosa cell apoptosis (as determined by the TUNEL assay) in the ovarian tissues from PCOS rats. In fact, studies have found that LH can inhibit the proliferation of granulosa cells, which would damage the growth and development of follicles [18]. Granulosa cells in the ovaries have decreased cell proliferation with degenerated granulosa cell layers in the testosterone-induced PCOS rats and human chorionic gonadotropin (hCG) plus *l*-norgestrel-induced PCOS rats [19], and an increased number of TUNEL (+) granulosa cells were found in dehydroepiandrosterone-induced PCOS rats [20]. In addition, studies also showed that the decreased expression of bcl-2 gene and increased expression of BAX gene and caspase-3 gene were detected in the PCOS rats [21]. The changes in the cell proliferation and apoptosis in the PCOS rats from our studies were consistent with previous studies. After the Diane-35 plus metformin treatment, the expression of PCNA was enhanced and the number of TUNEL-positive cells were reduced, implying that Diane-35 plus metformin could improve the ovarian function possibly via increasing granulosa cell proliferation and inhibiting granulomas cell apoptosis.

The normal development of follicles relies mainly on the uptake of glucose from surrounding tissues by granulosa cells through the GLUT on their cell membranes, and the production of pyruvate and lactic acid via the glycolysis pathway to provide energy for follicular development [22, 23]. Studies have found that the glycolytic activity was increased in developing follicles and as the diameter of the follicle increased, the rate of lactic acid production in the follicular fluid increased significantly [24]. In addition, the lactic acid content in the follicular fluid of PCOS patients was significantly lower than that of normal women [25]. Our study showed that the expression of GLUT1 was significantly increased in the ovary of PCOS rats, and the expression of HK and PFK was significantly decreased, suggesting that the utilization of glucose in PCOS rats was decreased. Moreover, the expression of MCT2/4 was down-regulated, and the transport rate of pyruvate and lactic acid decreased. ATP level and lactic acid concentration in the ovary of PCOS rats was significantly reduced, suggesting the disordered energy metabolism in the PCOS ovary. Importantly, combination of Diane-35 and metformin treatment significantly restored above changes in the PCOS rats. In the human studies, Diane-35 could increase the triglycerides levels, but had no relevant negative effects in the metabolic system in the PCOS patients, while Diane-35 plus metformin was effective in improving the metabolic profiles of PCOS patients [26]. Collectively, these results indicated that Diane-35 plus metformin can improve the energy metabolism of the ovary via regulating the glycolysis pathway.

In the glycolysis pathway, PKM2 and LDH-A are two key glycolysis-related rate-limiting enzymes. PKM2 can catalyze the irreversible transphosphorylation between phosphoenolpyruvate (PEP) and adenosine diphosphate, which produces pyruvate and ATP [27]. LDH-A can convert pyruvate into lactic acid by using NAD+ and NADH as coenzymes, rapidly regenerating NAD+ to maintain high-speed glycolysis [28]. Studies have demonstrated that increased glycolytic activity was associated with the up-regulation of PKM2 and LDH-A in the cumulus granulosa cells [29]. The abnormal expression of PKM2 can promote the proliferation, migration and invasion of tumor cells and other malignant biological behaviors [30]. In the ovary cancer, knockdown of PKM2 resulted in inhibition of tumor cell proliferation [31]. Recent studies by Wang et al., showed that PKM2 was down-regulated in the endometrial tissues from PCOS patients with hyperplasia, which was attenuated by metformin treatment [32]. Our data showed that PKM2 was down-regulated in the ovarian tissues from PCOS rats, which was attenuated by the Diane-35 plus metformin treatment. The results suggest that PKM2 plays an important role in the glycolysis of follicular energy metabolism and Diane-35 plus metformin could improve the PCOS glycolysis process. Studies have shown that the expression of LDH-A in granulosa cells is higher than that in oocytes [33], and the expression of LDH-A in COCs was remarkably increased in the late stage of follicular development [34]. Androgen could inhibit LDH-A expression to reduce lactic acid production, which has been suggested to be associated with PCOS follicular developmental disorders [35]. More importantly, excessive nerve growth factor in follicular fluid of PCOS patients can significantly reduce the expression of LDH-A, impair communication between granulosa cells and oocytes, and reduce oocyte developmental capacity [36]. Metformin treatment could increase the LDH-A expression in the endometrial tissues from PCOS patients with hyperplasia [32]. Furthermore, the interaction between LDH-A and SIRT1 could facilitate metabolic channeling and the subsequent epigenetic modification in the nucleus [37]. SIRT1 is a key NAD^+^-dependent deacetylase, which is regulated by nuclear levels of NADH, could regulate cell proliferation and apoptosis [38]. SIRT1 could regulate the gluconeogenic/glycolytic pathways in liver in response to maintain blood glucose levels [39], and activation of SIRT1 can significantly inhibit the ROS and NO production, and improve the oxidative stress-related insulin resistance [40]. More importantly, recent studies found that the expression of SIRT1 in the ovaries of PCOS rats was significantly decreased, which could be improved by metformin treatment [41, 42], and consistently, our data showed that SIRT1 was down-regulated in the ovary of POCS rats, which was restored the Diane-35 plus metformin treatment. Collectively, the therapeutic effects of Diane-35 plus metformin treatment in the PCOS rats may be associated with the regulation of glycolysis-related mediators (PKM2 and LDH-A) and SIRT1.

The present study for the first time demonstrated the beneficial effects of Diane-35 plus metformin treatment in PCOS rats, and more importantly, mechanistic studies revealed that several key mediators that related with glycolysis and energy metabolism could be modulated by the Diane-35 plus metformin treatment. However, the investigation is still limited to its preliminary stage, and further functional studies of these mediators in future studies should be investigated. As Diane-35 and metformin has been suggested to treatment PCOS, further clinical studies may be performed to determine the expression of these key mediators in the PCOS patients after the treatment, and to evaluate the clinical significance of these mediators.

## Conclusions

In conclusion, our study demonstrated that Diane-35 plus metformin treatment improved the pathological changes in the PCOS rats. Further studies suggest that Diane-35 plus metformin can improve the energy metabolism of the ovary via regulating the glycolysis pathway. The mechanistic studies indicated that the therapeutic effects of Diane-35 plus metformin treatment in the PCOS rats may be associated with the regulation of glycolysis-related mediators (PKM2 and LDH-A) and SIRT1. Future studies were required to determine the biological function of the glycolysis-related mediators in PCOS patients.

## Data Availability

Data are available upon request from the corresponding author.

## Abbreviations

DM: Diane-35 combined with metformin
FSH: Follicle-stimulating hormone
GLUT: Glucose transporter
HK: Hexokinase
LDH-A: Lactate dehydrogenase A
LH: Luteinizing hormone
MCT2: Monocarboxylate transporter 2
MCT4: Monocarboxylate transporter 4
PBS: Phosphate buffered saline
PCNA: Proliferating cell nuclear antigen
PFK: Phosphofructokinase
PKM2: Pyruvate kinase isozyme M2
PCOS: Polycystic ovary syndrome
SD: Sprague Dawley
SIRT1: Sirtuin 1
TUNEL: Terminal Deoxynucleotidyl Transferase-Mediated dUTP Nick End-Labeling

## References

[1] Garad R, Shorakae S, Teede H (2019). Assessment and Management of Women with polycystic ovary syndrome (PCOS).

[2] Chen M-J, Chou C-H, Chen S-U, Yang W-S, Yang Y-S, Ho H-N (2015). The effect of androgens on ovarian follicle maturation: Dihydrotestosterone suppress FSH-stimulated granulosa cell proliferation by upregulating PPARγ-dependent PTEN expression. Sci Rep.

[3] Diamanti-Kandarakis E, Dunaif A (2012). Insulin resistance and the polycystic ovary syndrome revisited: an update on mechanisms and implications. Endocr Rev.

[4] Dadachanji R, Shaikh N, Mukherjee S (2018). Genetic variants associated with Hyperandrogenemia in PCOS pathophysiology. Genet Res Int.

[5] Feng W, Jia YY, Zhang DY, Shi HR (2016). Management of polycystic ovarian syndrome with Diane-35 or Diane-35 plus metformin. Gynecol Endocrinol.

[6] Makanji Y, Tagler D, Pahnke J, Shea LD, Woodruff TK (2014). Hypoxia-mediated carbohydrate metabolism and transport promote early-stage murine follicle growth and survival. Am J Endocrinol Metab.

[7] Anastácio A, Rodriguez-Wallberg KA, Chardonnet S, Pionneau C, Fédérici C, Santos TA, Poirot C. Protein profile of mouse ovarian follicles grown in vitro. Mol Hum Reprod. 2017;23:827–41. 10.1093/molehr/gax056 PMC5909860 29069483

[8] Peralta OA, Bucher D, Angulo C, Castro MA, Ratto MH, Concha I (2016). Tissue localization of GM-CSF receptor in bovine ovarian follicles and its role on glucose uptake by mural granulosa cells. Anim Reprod Sci.

[9] Harris SE, Maruthini D, Tang T, Balen AH, Picton HM (2010). Metabolism and karyotype analysis of oocytes from patients with polycystic ovary syndrome. Hum Reprod.

[10] Zhao S, Xu H, Cui Y, Wang W, Qin Y, You L, Chan WY, Sun Y, Chen ZJ (2016). Metabolic actions of insulin in ovarian granulosa cells were unaffected by hyperandrogenism. Endocrine.

[11] Kafali H, Iriadam M, Ozardalı I, Demir N (2004). Letrozole-induced polycystic ovaries in the rat: a new model for cystic ovarian disease. Arch Med Res.

[12] Loo DT (2011). In situ detection of apoptosis by the TUNEL assay: an overview of techniques. Methods Mol Biol.

[13] Tosi F, Bonora E, Moghetti P (2017). Insulin resistance in a large cohort of women with polycystic ovary syndrome: a comparison between euglycaemic-hyperinsulinaemic clamp and surrogate indexes. Hum Reprod.

[14] Shorakae S, Ranasinha S, Abell S, Lambert G, Lambert E, de Courten B, Teede H (2018). Inter-related effects of insulin resistance, hyperandrogenism, sympathetic dysfunction and chronic inflammation in PCOS. Clin Endocrinol.

[15] Buzney E, Sheu J, Buzney C, Reynolds RV (2014). Polycystic ovary syndrome: a review for dermatologists: Part II. Treatment. J Am Acad Dermatol.

[16] Palomba S, Falbo A, Zullo F, Orio F (2009). Evidence-based and potential benefits of metformin in the polycystic ovary syndrome: a comprehensive review. Endocr Rev.

[17] Zhang F, Ma T, Cui P, Tamadon A, He S, Huo C, Yierfulati G, Xu X, Hu W, Li X (2019). Diversity of the gut microbiota in Dihydrotestosterone-induced PCOS rats and the pharmacologic effects of Diane-35, probiotics, and Berberine. Front Microbiol.

[18] Maman E, Yung Y, Kedem A, Yerushalmi GM, Konopnicki S, Cohen B, Dor J, Hourvitz A (2012). High expression of luteinizing hormone receptors messenger RNA by human cumulus granulosa cells is in correlation with decreased fertilization. Fertil Steril.

[19] Chen H, Guo JH, Zhang XH, Chan HC (2015). Defective CFTR-regulated granulosa cell proliferation in polycystic ovarian syndrome. Reproduction.

[20] Rencber SF, Ozbek SK, Eraldemır C, Sezer Z, Kum T, Ceylan S, Guzel E (2018). Effect of resveratrol and metformin on ovarian reserve and ultrastructure in PCOS: an experimental study. J Ovarian Res.

[21] Ding L, Gao F, Zhang M, Yan W, Tang R, Zhang C, Chen ZJ (2016). Higher PDCD4 expression is associated with obesity, insulin resistance, lipid metabolism disorders, and granulosa cell apoptosis in polycystic ovary syndrome. Fertil Steril.

[22] Boland NI, Humpherson PG, Leese HJ, Gosden RG (1994). Characterization of follicular energy metabolism. Hum Reprod.

[23] Cinco R, Digman MA, Gratton E, Luderer U (2016). Spatial characterization of bioenergetics and metabolism of primordial to Preovulatory follicles in whole ex vivo murine ovary. Biol Reprod.

[24] Boland NI, Humpherson PG, Leese HJ, Gosden RG (1994). The effect of glucose metabolism on murine follicle development and steroidogenesis in vitro. Hum Reprod.

[25] Zhang Y, Liu L, Yin TL, Yang J, Xiong CL (2017). Follicular metabolic changes and effects on oocyte quality in polycystic ovary syndrome patients. Oncotarget.

[26] Wu H, Ruan X, Jin J, Mueck AO (2015). Metabolic profile of Diane-35 versus Diane-35 plus metformin in Chinese PCOS women under standardized life-style changes. Gynecol Endocrinol.

[27] Wei L, Dai Y, Zhou Y, He Z, Yao J, Zhao L, Guo Q, Yang L (2017). Oroxylin A activates PKM1/HNF4 alpha to induce hepatoma differentiation and block cancer progression. Cell Death Dis.

[28] Min S, Jiujie C, Jiawei D, Daoyan W, Zhiliang J, Jun Z, Zhenggang Z, Yong G, Keping X (2014). A novel KLF4/LDHA signaling pathway regulates aerobic glycolysis in and progression of pancreatic cancer. Clin Cancer Res.

[29] Sugiura K, Pendola FL, Eppig JJ (2005). Oocyte control of metabolic cooperativity between oocytes and companion granulosa cells: energy metabolism. Dev Biol.

[30] Wang C, Jiang J, Ji J, Cai Q, Chen X, Yu Y, Zhu Z, Zhang J (2017). PKM2 promotes cell migration and inhibits autophagy by mediating PI3K/AKT activation and contributes to the malignant development of gastric cancer. Sci Rep.

[31] Miao Y, Lu M, Yan Q, Li S, Feng Y (2016). Inhibition of Proliferation, Migration, and Invasion by Knockdown of Pyruvate Kinase-M2 (PKM2) in Ovarian Cancer SKOV3 and OVCAR3 Cells. Oncol Res.

[32] Wang T, Zhang J, Hu M, Zhang Y, Cui P, Li X, Li J, Vestin E, Brannstrom M, Shao LR, Billig H (2019). Differential expression patterns of glycolytic enzymes and mitochondria-dependent apoptosis in PCOS patients with endometrial hyperplasia, an early Hallmark of endometrial Cancer, in vivo and the impact of metformin in vitro. Int J Biol Sci.

[33] Zhang L, Shao SM, Chen M. Expressions and significances of phosphofructokinase and lactatedehydrogenase associated with glycolytic pathway in human ovarian tissue. Matern Child Health Care China. 2014;9:1402–4.

[34] Lamas-Toranzo I, Pericuesta E, Bermejo-Álvarez P (2018). Mitochondrial and metabolic adjustments during the final phase of follicular development prior to IVM of bovine oocytes. Theriogenology.

[35] Liu Y, Liu X, Li D. Effect of testosterone on lactate production of granulose cells in mice. Jiangsu Med J. 2013;12:1398-1401.

[36] Zhai Y, Yao G, Rao F, Wang Y, Song X, Sun F (2018). Excessive nerve growth factor impairs bidirectional communication between the oocyte and cumulus cells resulting in reduced oocyte competence. Reprod Biol Endocrinol.

[37] Castonguay Z, Auger C, Thomas SC, Chahma M, Appanna VD (2014). Nuclear lactate dehydrogenase modulates histone modification in human hepatocytes. Biochem Biophys Res Commun.

[38] Braidy N, Guillemin GJ, Mansour H, Chanling T, Poljak A, Grant R (2011). Age related changes in NAD+ metabolism oxidative stress and Sirt1 activity in wistar rats. PLoS One.

[39] Rodgers JT, Carlos L, Wilhelm H, Gygi SP, Spiegelman BM, Pere P (2005). Nutrient control of glucose homeostasis through a complex of PGC-1alpha and SIRT1. Nature.

[40] Cao Y, Jiang X, Ma H, Wang Y, Xue P, Liu Y (2016). SIRT1 and insulin resistance. J Diabetes Complications.

[41] Tao X, Zhang X, Ge SQ, Zhang EH, Zhang B (2015). Expression of SIRT1 in the ovaries of rats with polycystic ovary syndrome before and after therapeutic intervention with exenatide. Int J Clin Exp Pathol.

[42] Tao X, Cai L, Chen L, Ge S, Deng X (2019). Effects of metformin and Exenatide on insulin resistance and AMPKalpha-SIRT1 molecular pathway in PCOS rats. J Ovarian Res.

